# Age-Specific Long-Term Effects Of Sleep On Memory And Executive Function: MIDUS 2 And 3

**DOI:** 10.1093/geroni/igaf122.3881

**Published:** 2025-12-31

**Authors:** Sunbok Park, Jeongok Logan, Ha Do Byon

**Affiliations:** University of Virginia School of Nursing, Charlottesville, Virginia, United States; University of Virginia School of Nursing, Charlottesville, Virginia, United States; University of Virginia School of Nursing, Charlottesville, Virginia, United States

## Abstract

Maintaining cognitive health during aging is critical, and sleep quality has emerged as a modifiable factor in cognitive decline. Yet, age-specific associations between sleep and long-term cognitive changes, independent of mental and neurological conditions, remain underexplored in nationally representative samples. Using data from the Midlife in the United States (MIDUS) study, sleep quality was assessed at MIDUS 2 with the Pittsburgh Sleep Quality Index (PSQI), and cognitive function was reassessed approximately 9.2 years later at MIDUS 3. Cognitive domains included episodic memory, executive functioning, and a composite score, standardized to MIDUS 2 means and standard deviations. Partial correlations examined associations between baseline sleep and cognitive change, controlling for age, sex, education level, psychiatric visits in the past 12 months, and neurological disorders in the past year from MIDUS 2. Analyses were stratified by age: older adults (≥65 years, n = 92) and young–middle-aged adults (< 65 years, n = 537). Among older adults, episodic memory decline was linked to poorer global sleep (r=-0.286, p = 0.005), longer sleep latency (r=-0.244, p = 0.018), and lower habitual sleep efficiency (r=-0.306, p = 0.003), with no associations for executive functioning. In contrast, among young–middle-aged adults, executive functioning decline was associated with poor subjective sleep quality (r=-0.116, p = 0.007) and shorter sleep duration (r=-0.113, p = 0.009). These findings highlight distinct age-specific vulnerabilities: episodic memory in older adults linked to structural sleep elements, and executive functioning in younger adults linked to subjective quality and duration. Tailored sleep interventions, emphasizing latency reduction and efficiency in older adults and duration/quality in younger adults, may help preserve long-term cognitive health.

